# A Single-Round Infection Fluorescent SARS-CoV-2 Neutralization Test for COVID-19 Serological Testing at a Biosafety Level-2 Laboratory

**DOI:** 10.3390/v14061211

**Published:** 2022-06-02

**Authors:** Jing Zou, Hongjie Xia, Pei-Yong Shi, Xuping Xie, Ping Ren

**Affiliations:** 1 Department of Biochemistry & Molecular Biology, University of Texas Medical Branch, Galveston, TX 77555, USA; jizou@utmb.edu (J.Z.); hoxia@utmb.edu (H.X.); peshi@utmb.edu (P.-Y.S.); 2 Institute for Human Infection and Immunity, University of Texas Medical Branch, Galveston, TX 77555, USA; 3 Sealy Institute for Drug Discovery, University of Texas Medical Branch, Galveston, TX 77555, USA; 4 Institute for Translational Sciences, University of Texas Medical Branch, Galveston, TX 77555, USA; 5 Sealy Institute for Vaccine Sciences, University of Texas Medical Branch, Galveston, TX 77555, USA; 6 Sealy Center for Structural Biology & Molecular Biophysics, University of Texas Medical Branch, Galveston, TX 77555, USA; 7 Department of Pathology, University of Texas Medical Branch, Galveston, TX 77555, USA

**Keywords:** single-round infection fluorescent SARS-CoV-2 virus, neutralization assay, sensitivity, specificity, accuracy, linearity

## Abstract

A robust serological test to measure neutralizing antibodies against SARS-CoV-2 in biosafety level-2 (BSL-2) laboratories is useful for monitoring antibody response after vaccination or natural infection. The gold standard assay is the conventional plaque reduction neutralization test (PRNT) which requires extensive labor, live viruses, and BSL-3 facilities. Recently, we developed a novel single-round infection fluorescent SARS-CoV-2 virus (SFV) that can be safely used at BSL-2 laboratories for high-throughput neutralization and antiviral testing. In this study, we evaluated the performance of the neutralization test using this SFV with 80 PRNT-positive and 92 PRNT-negative clinical serum or plasma specimens. The SFV neutralization test (SFVNT) has 100% sensitivity and specificity compared to the PRNT. Furthermore, the neutralizing titers generated by the SFVNT and PRNT are highly correlated, with R^2^ = 0.903 (*p* < 0.0001). Due to high sensitivity, specificity, accuracy, and reproducibility, the SFVNT can be deployed for the large-scale testing of COVID-19 patients or vaccinated people in general lab settings.

## 1. Introduction

Severe acute respiratory syndrome coronavirus 2 (SARS-CoV-2) has caused the coronavirus disease (COVID-19) pandemic. Diagnostic testing for SARS-CoV-2 infection is accomplished by detecting viral RNA, which is known as molecular testing, or by detecting viral proteins via antigen assays. At the same time, serological IgM testing can detect current or recent infections. Positive IgG may indicate past exposure or infection or vaccination status for surveillance and research purposes. To combat the COVID-19 pandemic, vaccine and antiviral drug developments play crucial roles. It is important to measure the neutralizing antibody levels after vaccination, therapeutic antibody treatment, and natural infections because neutralizing antibodies are among the most protective immune parameters. The conventional plaque reduction neutralization test (PRNT) is the gold standard serological assay to quantify the neutralizing antibody levels. However, the PRNT remains labor-intensive, time-consuming and has low throughput. It also requires infectious SARS-CoV-2 pathogens and biosafety level-3 (BSL-3) facilities. Therefore, it is not accessible to many researchers, which creates a significant gap for COVID-19 surveillance, vaccine development, and therapeutic antibody testing. 

We previously developed a stable mNeonGreen (mNG, a green fluorescence protein) SARS-CoV-2 (icSARS-CoV-2-mNG) where the mNG gene was engineered at the ORF7 of the viral genome [1]. There was no transcriptional defect associated with the deletion of ORF7a/7b [2]. The icSARS-CoV-2-mNG reporter virus created from the reverse genetic system allows the use of fluorescence (mNG) as a surrogate readout for viral replication. The previous study [1] also demonstrated that the stability of the mNG reporter virus allows it to be used for longer-term studies and in vivo without fear of losing its fluorescent marker. The mNG reporter SARS-CoV-2 was used to develop a neutralization test [3] similar to the PRNT in principle. Both assays quantify neutralizing antibody titers. Importantly, the mNG SARS-CoV-2 neutralizing assay (mNG-NT) uses mNG-tagged SARS-CoV-2 and quantifies neutralizing antibody titers within 24 h; in contrast, the PRNT usually requires 2 to 3 days to form visible SARS-CoV-2 plaques on Vero E6 cells. It significantly shortens the turnaround time of the assay. In addition, mNG-NT is performed in a 96-well-plate format, which enables higher throughput. However, the BSL-3 requirement prevents the mNG-NT from wide use. 

More recently, we developed a novel single-round infection fluorescent SARS-CoV-2 virus (SFV) that can be safely used at the BSL-2 for high-throughput neutralization and antiviral testing [4]. Based on the mNG SARS-CoV-2 virus genome, ORF3 and envelope E genes were removed (ΔORF3-E mNG) since the deletion of viral ORF3 and E, as well as the trans-complementation of the deleted proteins, have been reported for live-attenuated vaccine development for coronaviruses [5]. Through *trans*-complementation in the Vero E6–ORF3-E cells (cells express ORF3 and E proteins), high titers of SFV (ΔORF3–E mNG virions) can be generated. This system produces single-round infectious ΔORF3–E mNG virions that infect normal cells just once. Thus, it cannot spread *in vitro* or *in vivo* since normal cells do not express viral ORF3 and E proteins which are required for SARS-CoV-2 assembly. Most importantly, SFV recapitulates authentic viral replication without virulence, which enables us to manipulate the viruses at BSL-2. In this study, we evaluated the performance characteristics of the neutralization test (SFVNT) using this novel SFV and compared it to the gold standard PRNT using clinical serum/plasma specimens. The sensitivity, specificity, accuracy, linearity, and reproducibility of the SFVNT were investigated. 

## 2. Materials and Methods

### 2.1. Serum/Plasma Specimens

A total of 80 leftover clinical serum/plasma specimens for the routine standard of care were collected from COVID-19 vaccinated individuals. Another 92 serum/plasma specimens used in this study were collected before the emergence of COVID-19. 

### 2.2. Cells 

Vero–ORF3-E cells were maintained in high-glucose Dulbecco’s modified Eagle’s media (DMEM) supplemented with 2 mM L glutamine, 100 U/mL penicillium–streptomycin (P/S), 10% fetal bovine serum (FBS; HyClone laboratories, South Logan, UT, USA), 0.075% sodium bicarbonate, and 10 µg/mL puromycin. A549-hACE2 cells were kindly provided by Dr. Shinji Makino at UTMB [6] and grown in the DMEM media supplemented with 10 µg/mL blasticidin and 10 mM HEPES at 37 °C with 5% CO_2_. All media and other supplements were purchased from Thermo Fisher Scientific (Waltham, MA, USA). Cells tested mycoplasma negative. 

### 2.3. Preparation of SFV Stocks 

The initial stocks of SFV were generated as described previously [4]. Large stocks (P11) were prepared by passaging the tenth passage of SFVs on the Vero–ORF3-E cells with an MOI of 0.05. At 64 h post-infection, supernatants were clarified by centrifugation at 1000× *g* 4 °C for 10 min. Virion infectivity was quantified by measuring the TCID_50_ using an end-point dilution assay as previously reported [4]. Briefly, Vero–ORF3-E cells were plated on 96-well plates (1.5 × 10^4^ per well) one day before infection. SFV virions were serially diluted in DMEM supplemented with 2% FBS, with six replicates per concentration. Cells were infected with 100 µL of diluted virions and incubated at 37 °C for 3 days. The mNG-positive wells were counted under a fluorescence microscope (Nikon, Tokyo, Japan) and TCID_50_ was calculated using the Reed and Muench method. The viral genome sequences of the stocks were confirmed by Sanger sequencing to avoid any undesired mutations. 

### 2.4. SFV Neutralization Assay (SFVNT) 

A549-hACE2 (1.2 × 10^4^) in 50 µL culture medium with 2% FBS was seeded in black µClear flat-bottom 96-well plates (Greiner Bio-One). The next day, 30 µL of two-fold serial diluted human serum/plasma was mixed with an equal volume of SFVs (MOI of 0.5–3.3) and incubated at 37 °C for 1 h. Afterward, 50 µL of virus serum/plasma mixtures was added to each well of the black plates. After incubating at 37 °C with 5% CO_2_ for 16–24 h, 25 µL of Hoechst 33342 solution (400-fold diluted in Hank’s balanced salt solution; Thermo Fisher Scientific, Waltham, MA, USA) was added to each well to counterstain the cell nuclei. After an additional 10 min of staining, the mNG-positive cells were scanned using the CellInsight CX5 high-content screening platform (Thermo Fisher Scientific) with predefined threshold parameters obtained using non-infected and infected cells. The positive cells in each well were counted and normalized to the number of total cells, resulting in the infection rate. The infection rate from each well was finally normalized to the non-serum/plasma-treated controls to calculate the relative infectivities. The curves of the relative infectivity versus the serum/plasma dilutions (log_10_ values) were plotted using Prism 9 (GraphPad). A nonlinear regression method with a log (inhibitor) vs. response variable slope (four parameters) model (bottom and top parameters were constrained to 0 and 100, respectively) was used to determine the dilution fold that neutralized 50% of ΔORF3–E mNG SARS-CoV-2 (defined as SFVNT_50_) in GraphPad Prism 9. 

### 2.5. Plaque Reduction Neutralization Test (PRNT) 

The 50% plaque-reduction neutralization titer (PRNT_50_) was measured for the serum/plasma as previously reported [3,7]. Individual serum/plasma was two-fold serially diluted in culture medium with a starting dilution of 1:20. The diluted samples were incubated with 100 PFU of USA-WA1/2020. After 1 h of incubation at 37 °C, the serum/plasma virus mixtures were inoculated onto six-well plates with a monolayer of Vero E6 cells pre-seeded on the previous day. The PRNT_50_ value was defined as the minimal serum/plasma dilution that suppressed >50% of viral plaques. The neutralization titer was determined in duplicate assays, and the geometric mean was taken. 

### 2.6. Linearity Analysis 

To evaluate the linearity of the SFVNT, five neutralization-positive serum/plasma samples were 1:2 serially diluted five times. Each dilution was treated as an individual specimen and blinded for measuring neutralizing activities by the SFVNT as described above. 

### 2.7. Reproducibility 

Intra- and inter-reproducibility of the SFVNT were evaluated. First, the SFVNT was performed with four neutralization-positive and four negative serum/plasma samples with duplication in three different assay plates by the same analyst on two different days (assay I and II). Second, 10 negative and 48 positive human serum/plasma samples were used to perform the SFVNT using two different operators. The statistic differences were analyzed by a Wilcoxon matched-pairs signed-rank test. A *p*-value of less than 0.05 is treated as statistically significant. 

### 2.8. Ethics Statement 

This study was conducted under a research protocol (protocol 20-0070) approved by the institutional review board at the University of Texas Medical Branch (UTMB). The manipulation of SFV at BSL-2 labs has been approved by the institutional biosafety committee at UTMB and the Office of Science Policy from NIH. 

## 3. Results

### 3.1. Rationale and Workflow

**Figure 1:**
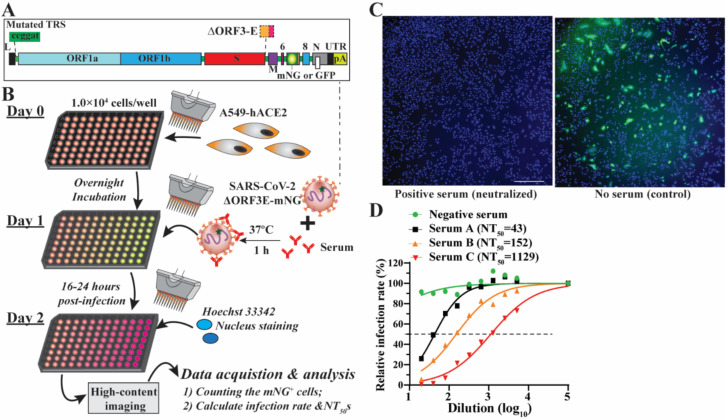
Rationale and workflow of the SFVNT assay. (**A**) The genome architecture of SFV. TRS, transcription regulation sequence; L, leader sequence; ORF, open reading frame; S, spike glycoprotein; E, envelope protein; M, membrane protein; N, nucleoprotein; UTR, untranslated region; mNG, mNeonGreen. (**B**) Workflow of the SFVNT. Details are described in the Section 2. (**C**) Representative fluorescence images of infected cells in the presence and absence of positive serum. Nuclei stained by Hoechst 33342 are shown in blue. mNG-positive cells are shown in green. (**D**) Neutralization curve of four sera with no, low, medium, and high neutralizing activities. SFVNT_50_s are indicated.

A single-round infection fluorescent SARS-CoV-2 virus (SFV) was developed to enable high-throughput neutralization and antiviral testing at BSL-2 laboratories [4]. SFVs were generated in a producer cell line (Vero E6–ORF3-E) expressing ORF3 and envelope (E) genes through the trans-complementation of a viral genomic RNA lacking these two genes (Figure 1A). mNG fluorescence protein was genetically engineered to enable the fast tracking of the virus in a real-time manner. Further, we developed an SFV-based neutralization test (SFVNT) to measure the neutralizing antibody titers in the 96-well plate format. Figure 1B outlines the workflow of the SFVNT on A549-hACE2 cells, a human lung epithelial cell line expressing human angiotensin-converting enzyme 2 (hACE2). Specifically, serum or plasma samples were two-fold serial diluted in a 96-well plate and incubated with SFV at 37 °C for 1 h. The serum/plasma-virus mixtures were added to the cells. After 16 h of infection, mNG fluorescence signals were detected in the SFV-infected cells. The total cell numbers were evaluated by nucleus counterstaining. The infected cells were counted using a high-content imager. The layout of the assay can be landscape or portrait, which allows for analyzing four (eleven two-fold serial doses with the control) or six samples (seven two-fold serial doses with the control) in duplicates. As shown in Figure 1C, SARS-CoV-2-positive serum prevented the A549-hACE2 cell from SFV infection and did not result in mNG-positive cells (left panel); in contrast, without serum treatment, SFV produced robust mNG fluorescence in the targeted cells (right panel). The inhibition of SFV depends on the dilution of the sample. Using a nonlinear regression mathematical model, the dilution fold that reduces 50% of SFV-infected cells (SFVNT_50_) can be assessed. Therefore, the neutralizing activities of human serum/plasma can be quantified and compared. For example, the dose-response curves of four serum/plasma samples are shown in Figure 1D, representing the corresponding serum/plasma samples with no, low, medium, and high SFV-neutralizing activities. Thereafter, we performed a comprehensive evaluation of the SFVNT to deploy it as a serological assay for potential clinical use. 

### 3.2. Optimization of the SFVNT

**Figure 2:**
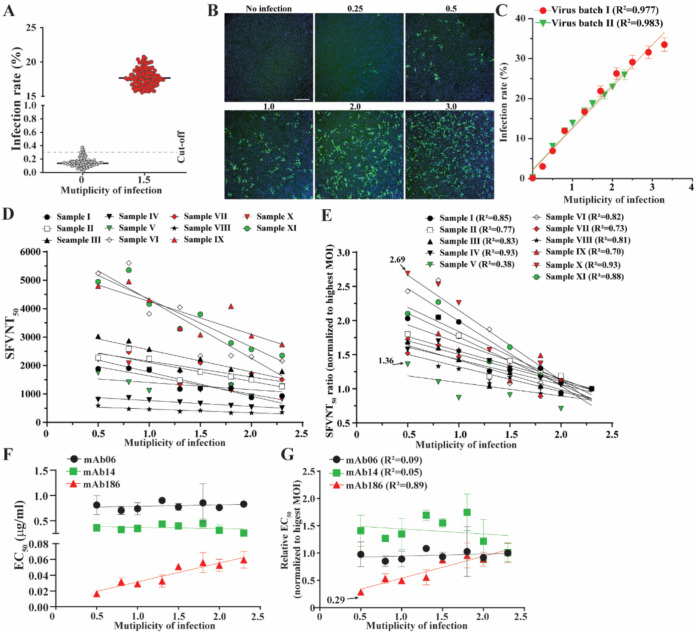
Optimization of MOIs for the SFVNT. (**A**) Background of autofluorescence from uninfected cells. A total of 120 wells of uninfected or infected (MOI of 1.5) A549-hACE2 cells were scanned by high-content imaging. Infection rates were calculated. The dotted line indicates the mean of the infection rate plus two times the standard deviation from uninfected wells. (**B**) Representative fluorescence images of A549-hACE2 cells infected with various amounts of input viruses. Numbers above each panel indicate the multiplicity of infection (MOI). (**C**) Correlation between MOIs and the infection rate. Two virus batches were tested. Pearson’s correlation coefficients are shown. *p* values (two-tailed) < 0.0001. (**D**) The correlation between the MOI and the SFVNT_50_ values. Eleven neutralization-positive samples were evaluated by the SFVT with various amounts of input viruses (MOI from 0.5 to 2.3). (**E**) The correlation between the MOI and normalized SFVNT_50_ values. For each sample, the SFNVT_50_ values were normalized to that obtained from an MOI of 2.3. (**F**) The correlation analysis of MOI and SFVNT_50_ values of three monoclonal antibodies. (**G**) The correlation analysis of MOI and normalized SFVNT_50_ values of three monoclonal antibodies. SFNVT_50_ ratios were calculated by normalizing each SFVNT_50_ value to that obtained from an MOI of 2.3. Linear regression correlation coefficients are shown.

The SFVNT involves two critical steps: the formation of virus serum/plasma mixtures and the infection of the targeting cells. The amount of input virus would affect both steps. Therefore, we optimized the SFVNT by determining the amount of input SFVs. Nonspecific signals always existed in high content imaging due to auto-fluoresces from the environmental dust, plastics, dead cells, salt, sample precipitants, etc. We defined the positive signal cut-off value as the mean plus two times of standard deviations of the infection rate obtained from 120 random wells with non-infected cells. The current protocol of the SFVNT gave a cut-off value of 0.3% (Figure 2A). In contrast, infection with a multiplicity of infection (MOI) of 1.5 yielded an average infection rate of 18%, corresponding to the signal-to-noise ratio of 60. Next, we determined the optimal amount of input virus by assessing the infection rates with various MOIs. As expected, the mNG-positive cells increased proportionally with the increasing numbers of input viruses (Figure 2B). The quantitative analysis revealed that the input virus and infection rate were highly correlated when the MOI was below 2.5. However, the correlation of the input virus and infection rate became nonlinear when the MOI was over 2.5 (Figure 2C), indicating the excess of input virus over the amount of hACE2 receptors for infection. Two different batches of stock viruses showed similar results (Figure 2C), indicating the high consistency of infection by SFV. Second, we measured the neutralization titers (SFVNT_50_) of serum/plasma with different amounts of input viruses. Eleven SFVNT-positive serum/plasma samples with known SFVNT_50_ values from 500 to 5000 were tested at different MOIs. As shown in Figure 2D,E, SFVNT_50_ generally increased as the MOI decreased in all test samples. Interestingly, 10 out of 11 sera exhibited a high correlation between SFVNT_50_ and MOI values except for Sample V. We hypothesized that the varied correlation between SFVNT_50_ and the MOI may be due to the heterogeneity of the neutralizing antibodies present in the samples. To further test our hypothesis, we examined three highly potent monoclonal antibodies, mAb6, mAb14 [8], and mAb186 (the manuscript was submitted for publication), with different binding epitopes in the receptor-binding domain of SARS-CoV-2. mAb186 showed a linear response of median effective concentrations (EC_50_) to the MOIs, while mAb06 and mAb14 showed constant EC_50_ values at all tested MOIs ranging from 0.5 to 2.3 (Figure 2F,G). Collectively, our data suggested that the number of input viruses would influence the assay’s sensitivity because of the antibody’s complexity or mode of action in the serum/plasma, although the extract mechanisms remain elucidated. Thus, caution should be taken when neutralizing titers are compared among multiple assays with different conditions. We finally chose the MOI of 1.5 in all the SFVNTs described below because it resulted in a robust infection rate (~18%) (Figure 2C) and values of neutralizing antibody titers similar to the conventional PRNT. The discrepancy between an MOI of 1.5 and 18% infection rate was likely caused by the different cells used to determine the MOI (on Vero E6–ORF3-E cells) and the infection rate (on A549-hACE2 cells).

### 3.3. Accuracy of the SFVNT

The accuracy of the SFVNT was evaluated using clinical serum/plasma specimens by comparing it to the conventional PRNT results. To obtain clean negative and positive results, the samples used in this study were collected from two different periods, before the emergence of COVID-19 in November 2019 and after the pandemic started. The samples collected after the pandemic started were from COVID-19 vaccinated individuals. All 92 negative serum/plasma samples collected before the pandemic had a PRNT_50_ of <20, which is considered negative. In agreement with the PRNT_50_ results, the SFVNT_50_ data were also negative. All 80 COVID-19 vaccinated samples showed a PRNT_50_ of 20 to 7241 and an SFVNT_50_ of 18 to 8913 (Table 1). All these vaccinated specimens qualitatively tested positive by both PRNT and SFVNT. Thus, the SFVNT exhibited 100% sensitivity (95% confidence interval (CI): 95.5–100%) and specificity (95% CI: 96.1–100%) when compared to the PRNT. 

### 3.4. Correlation between the SFVNT and PRNT

**Figure 3:**
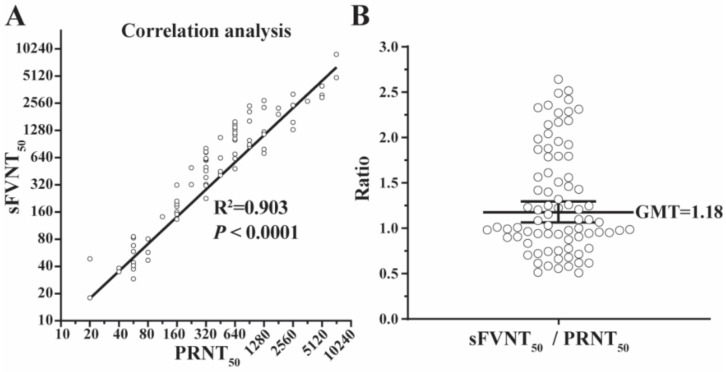
The correlation of the SFVNT_50_ versus PRNT_50_. (**A**) The correlation between the SFVNT_50_ and the conventional PRNT_50_. Eighty human serum/plasma samples were tested by the SFVNT and PRNT, respectively. Pearson’s correlation coefficient and P-value (two-tailed) are indicated. (**B**) The ratio of the SFVNT_50_ to PRNT_50_. The geometric mean (GM) of the ratios from 80 samples is indicated. The error bar shows the 95% confidence interval of GM.

The geometric means of titers (GMTs) generated by the SFVNT and PRNT are listed in Table 1 and plotted in Figure 3 for all 80 positive samples. The R^2^ between the two assays’ GMTs was 0.903 (*p* < 0.0001) by linear regression analysis (Figure 3A) and the GMT ratio of SFVNT_50_ to PRNT_50_ was 1.18 (Figure 3B), demonstrating that SFVNT_50_ correlated well with PRNT_50_. 

### 3.5. Linearity 

**Figure 4:**
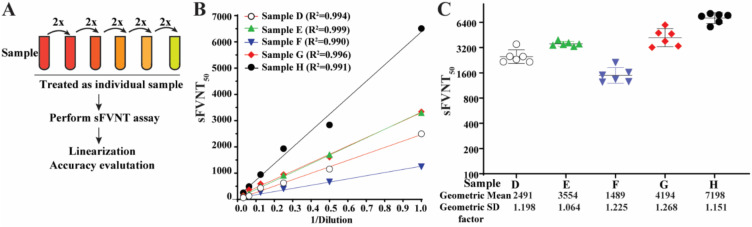
Linearity of the SFVNT. (**A**) The diagram of the workflow. Each neutralization-positive serum/plasma was two-fold serial diluted in a culture medium. Each dilution was treated as a sample and blinded for the SFVNT. (**B**) Person’s correlation coefficient between the dilution before the SFVNT and SFVNT_50_. *p* values (two-tailed) < 0.001. (**C**) The scatter-plot of the SFVNT_50_ values of five serum/plasma samples calculated from all six tests. The geometric mean and geometric standard deviation (SD) factors are indicated. The error bars show the 95% confidence intervals.

To determine the titer linearity of the SFVNT, SFVNT_50_ was measured from the serial dilutions of five specimens with known high neutralization titers (Figure 4A). The linear regression lines fit the titer values from the same original samples (Figure 4B). The R^2^ values were within the ranges of 0.990 to 0.999 (*p* < 0.001). The titers of each dilution from the same original samples were also plotted in their groups after adjusting the dilution factors (Figure 4C). The standard deviations for each group were between 1.064 to 1.268. 

### 3.6. Analytical Specificity

The analytical specificity of the SFVNT was evaluated with patient serum/plasma samples with antibodies and interfering substances that commonly exist in the blood. Two groups of specimens were included for cross-reactivity testing (Table 2). Group 1 included 135 clinical serum/plasma specimens from patients with antigens or antibodies against different viruses, bacteria, fungi, and parasites. They were manifested by positive diagnostic results for antibodies against specific pathogens (e.g., anti-cytomegalovirus; this group of samples is indicated by the prefix “anti” in Table 2), or positive diagnostic results for pathogen antigens or nucleic acids (e.g., *Cryptococcus neoformans* antigen or human coronavirus 229E, respectively; this group of samples is not indicated by a prefix in Table 2). Group 2 consisted of 32 samples with albumin, elevated bilirubin, cholesterol, rheumatoid factor, and some other antibodies that exist in the blood for active syphilis or autoimmune diseases. None of these specimens cross-neutralized SFVs (Table 2), including the four common cold coronaviruses (229E, HUK1, NL63, and OC43). 

### 3.7. Reproducibility 

**Figure 5:**
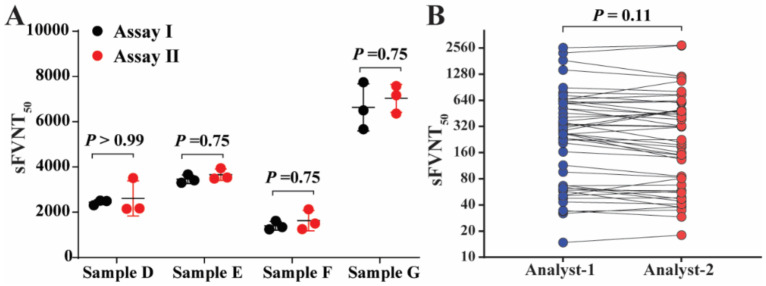
Reproducibility of the SFVNT. (**A**) Intra-reproducibility. The SFVNT was performed twice on different days using four neutralization-positive and four neutralization-negative samples tested in a duplication manner in three different plates. The mean and standard deviations from both repeats are shown (neutralization-negative samples are not shown). *p* values from the Wilcoxon matched-pairs signed-rank test are shown. (**B**) Inter-reproducibility. Ten negative and 48 positive serum/plasma samples were tested using the SFVNT by two different analysts. The SFVNT50 values for each neutralization-positive sample were compared. The *p* value from the Wilcoxon matched-pairs signed-rank test is shown.

To assess the intra-reproducibility, SFVNT was performed using four negative and four positive samples in duplicates in three different assay plates on two different days (Assay I and II) by the same operator (Figure 5A). All the results matched the expected qualitative results. The differences in the titers between two days of operation for all four positive samples were not significant (*p* > 0.05) (Figure 5A). To assess the inter-reproducibility, SFVNT was performed by two operators with 10 negative and 48 positive samples. Not only the qualitative results were matched, but there were also no significant differences between titers for the positive samples by two analysts (*p* > 0.05) (Figure 5B). The results indicated that the SFVNT achieved intra- and inter-assay precision. 

## 4. Discussion

In this study, we evaluated a rapid fluorescence-based high-throughput assay performed at a BSL-2 laboratory using the novel single-round infection viruses to measure the neutralizing antibody titers against SARS-CoV-2. Since the PRNT remains the gold standard for serological testing, we used the PRNT results as the reference to validate the SFVNT qualitatively and quantitatively. Similar to the PRNT, the SFVNT is a cell-based assay, which requires multi-factor optimization, for example, with cell types and the amount of virus input. The A549-hACE2 cell has been chosen for a higher susceptibility to SARS-CoV-2 infection than Vero E6 cells. A relatively high MOI (MOI of 1.5) was finally chosen for achieving better signal robustness and equivalent neutralization titers between the SFVNT and PRNT. Using these optimized conditions, we have systematically assessed the performance of the SFVNT. 

There was no discrepancy between the SFVNT and PRNT in terms of whether the specimens contained the SARS-CoV-2-neutralizing antibody or not. Data showed 100% matches for the positives and negatives between the two assays. Meanwhile, SFVNT generated NT_50_ values comparable to the conventional PRNT for the positive samples (R^2^ = 0.903, *p* < 0.0001). Compared with the PRNT assay, the SFVNT shortened the turnaround time by several days and increased the testing capacity to high throughput. 

Another attractive surrogate system to measure neutralizing antibodies at BSL-2 laboratories for vaccine and antibody development [4,9] is SARS-CoV-2 pseudovirus that expresses and presents SARS-CoV-2 spike proteins on the surface of a non-coronavirus [10,11], such as human immunodeficiency virus (HIV-1) [12], murine leukemia virus [13], or vesicular stomatitis virus [14]. However, several intrinsic limitations of the SARS-CoV-2 pseudovirus should be considered. First, the entry mechanisms of the pseudovirus may differ from authentic SARS-CoV-2 infection due to the lack of other SARS-CoV-2 structural proteins including membrane, envelope, and nucleocapsid proteins [15]. Second, the pseudovirus and authentic SARS-CoV-2 use different cellular compartments for budding and egress. The distribution, abundance, or displaying of S proteins on the pseudovirus may be different from the authentic SARS-CoV-2. In contrast, SFV not only has the feature of being used at BSL-2 facilities but also likely reflects the “natural” status of spike proteins appearing in the authentic SARS-CoV-2 virus, because SFV virions are egressed and matured with all the structural components through an extract mechanism as an authentic SARS-CoV-2 infection. Therefore, the SFVNT can precisely mimic the authentic SARS-CoV-2 infection, which could be an advantage when being used to measure neutralizing antibodies. In addition, due to the high similarities of SFV with authentic viruses, SFV may have additional applications in the biology of SARS-CoV-2 at BSL-2 labs, such as studying the mechanisms of cell-to-cell transmission *in vitro* [16,17]. 

In addition to mNG, other reporter genes, such as GFP and luciferase, can also be engineered into the single-round infection system. Different reporter genes may be tailored to support different project aims. For example, a luciferase reporter system can yield a more dynamic assay range than the mNG reporter system for screening compound libraries. Compared with the well-established micro-neutralization assay [18], our PRNT assay is homogenous (i.e., without wash steps) and does not require BSL-3. In addition, our single-round infection system can be easily adapted to test neutralization against newly emerged variants. This can be accomplished by generating new *trans*-complementing cell lines expressing the spike genes from variants of interest. A similar approach has been used to generate authentic SARS-CoV-2 bearing different variant spike proteins for testing antibody neutralization [19,20]. However, there are several limitations about this assay. It requires ΔORF3–E mNG SARS-CoV-2 stock virus and ORF3–E-expressing cells that are not commercially available. The fluorescent image system used for scanning mNG-positive cells could be expensive. These weaknesses may limit its application to general clinical laboratories. 

In summary, the current study has validated the potential use of a fluorescent single-round infection assay for the neutralization testing of clinical specimens at BSL-2. The results indicate the new SFVNT retains the gold standard of the PRNT assay but significantly shortens the assay turnaround time and improves the assay throughput. There are ongoing efforts to deploy the SFVNT assay for large-scale clinical specimen testing.

## Figures and Tables

**Table 1:** Comparison of neutralization titers of vaccinated subjects’ sera/plasmas analyzed by the PRNT and SFVNT.

Serum ID	^a^ PRNT_50_	^b^ SFVNT_50_	Serum ID	^a^ PRNT_50_	^b^ SFVNT_50_
1	20	18	41	453	1067
2	20	48	42	453	633
3	40	38	43	453	449
4	40	35	44	640	481
5	57	83	45	640	624
6	57	85	46	640	1000
7	57	37	47	640	700
8	57	58	48	640	1307
9	57	29	49	640	1491
10	57	44	50	640	1031
11	57	68	51	640	1143
12	57	41	52	640	1203
13	80	47	53	640	1389
14	80	57	54	640	693
15	80	81	55	640	1592
16	113	141	56	905	1623
17	160	185	57	905	993
18	160	318	58	905	850
19	160	196	59	905	2057
20	160	160	60	905	2391
21	160	133	61	905	892
22	160	151	62	1280	793
23	160	150	63	1280	1205
24	160	211	64	1280	1233
25	226	321	65	1280	1162
26	226	495	66	1280	713
27	320	499	67	1280	2296
28	320	731	68	1280	2741
29	320	457	69	1810	2255
30	320	323	70	1810	1929
31	320	598	71	2560	2434
32	320	225	72	2560	1303
33	320	314	73	2560	1573
34	320	615	74	2560	3222
35	320	806	75	3620	2695
36	320	386	76	5120	3975
37	320	740	77	5120	3151
38	320	626	78	5120	2962
39	453	441	79	7241	4911
40	453	410	80	7241	8913

^a^ PRNT_50_ values were the geometric means of titers (GMTs) derived from two independent plaque reduction neutralization assays (PRNT) using recombinant SARS-CoV-2 on Vero E6 cells. ^b^ SFVNT_50_ values were the GMTs derived from two independent neutralization assays using ΔORF3–E mNG SARS-CoV-2 on A549-hACE2 cells.

**Table 2:** Cross-reactivity of the single-round infection fluorescent SARS-CoV-2 virus (ΔORF3–E mNG SARS-CoV-2) neutralization test (SFVNT).

^a^ Immune Sera and ^b^ Interfering Substances	Sample Number	Number of SFVNT-Positive
Adenovirus	1	0
*Cryptococcus neoformans* antigen	1	0
Anti-cytomegalovirus	10	0
Anti-Epstein–Barr virus capsid or nuclear antigen	15	0
Anti-hepatitis A virus	9	0
Anti-hepatitis B virus surface or core antigen	21	0
Anti-hepatitis C virus	7	0
Anti-herpes simplex virus 1	8	0
Anti-herpes simplex virus 2	3	0
Human coronavirus 229E	1	0
Human coronavirus HKU1	2	0
Human coronavirus NL63	1	0
Human coronavirus OC43	3	0
Anti-human immunodeficiency virus 1	4	0
Human rhinovirus	2	0
Influenza B virus	1	0
Anti-measles virus	2	0
Anti-mumps virus	1	0
Respiratory syncytial virus	1	0
Anti-rubella virus	16	0
Anti-varicella zoster virus	20	0
Anti-West Nile virus	5	0
Anti-yellow fever virus (vaccination)	1	0
^b^ Albumin (4.5–4.9 g/dL)	7	0
^b^ Elevated total bilirubin (1.1 mg/dL)	5	0
^b^ Elevated cholesterol (>200 mg/dL)	3	0
^b^ Elevated rheumatoid factor (>100 IU/mL)	4	0
^b^ Anti-cardiolipin	2	0
^b^ Anti-mitochondrial M2	1	0
^b^ Anti-nuclear antibodies	10	0

^a^ A total of 135 serum/plasma specimens with antigens or antibodies against different infections (or immunizations) were tested against the SFVNT. They are listed in alphabetical order. Samples that tested positive for antibodies against specific pathogens are indicated with the prefix “anti”, whereas samples that tested positive for antigens or pathogen nucleic acids are not indicated with a prefix. For the latter group, the specimens were collected within 1 to 6 months after they PCR tested positive for pathogens. ^b^ A total of 32 samples tested for interfering substances, antibodies for active syphilis, and some autoimmune diseases.

## Data Availability

The original contributions presented in the study are included in the article. Further inquiries can be directed to the corresponding authors.
